# Draft Genome Sequence of Bacillus coagulans ZB29, a Commercial Probiotic Strain

**DOI:** 10.1128/MRA.01125-18

**Published:** 2019-01-17

**Authors:** Darshana Chauhan, Yama Patel, Jayraj A. Doshi, Rashesh D. Doshi, Anjali Bose

**Affiliations:** a Strain Management Department, Zytex Biotech Private Limited, Vadodara, Gujarat, India; University of California, Riverside

## Abstract

Bacillus coagulans ZB29, isolated from milk, is a safe strain already available on market shelves and characterized by certified beneficial effects. Here, we report the 3,646,473-bp genome sequence of this bacterium, its sequence assembly, and its annotations.

## ANNOUNCEMENT

Bacillus coagulans, formerly known as Lactobacillus sporogens, is emerging as a promising probiotic candidate owing to its good prophylactic properties and enhanced shelf life. However, the probiotic attributes and health-promoting properties are strain specific and cannot be generalized.

B. coagulans ZB29 was isolated from milk collected from the Mangal Dairy in Santacruz, Mumbai. The pure culture of ZB29 was maintained on glucose yeast extract (GYE) agar slants at 4°C. For genome sequencing, a single isolated colony was picked from a freshly grown plate and cultured in GYE broth overnight at 37°C under aerobic conditions. Total genomic DNA was isolated and purified using a MasterPure Gram-positive DNA purification kit (Epicentre, Cambio Ltd., Cambridge, UK). The isolated DNA was quantified by fluorescence with Qubit technology. Purity was assessed spectrophotometrically by determining the A_260_/A_280_ ratio. This DNA was fragmented using a Bioruptor NGS ultrasonicator (Diagenode, Inc., NJ). The sequencing library was constructed employing the TruSeq DNA PCR-free library prep kit (Illumina, Inc., CA) and using 2.5 μg of genomic DNA. Library samples were loaded onto a flow cell with MiSeq reagent v2 for 500 cycles (Illumina, Inc., CA) according to the manufacturer’s technical guide and were sequenced as paired-end reads (2 × 250 bp) using the Illumina Miseq sequencing platform. A total of 939,917 quality-approved paired-end reads were *de novo* assembled into 170 contigs (113.31× coverage, *N*_50_ value of 48,317 bp) with a total genome size of 3,639,373 bp using the MIRA v4.0.2 assembler (1) in “accurate” mode coupled with the parameters “genome” and “denovo.” These assembled contigs were anchored onto the nearest reference genome of *B. coagulans* 36D1 (NCBI reference sequence number NZ_CP009709) using the bacterial genome finishing tool MeDuSa v1.6 (2) to yield the final genome (3,646,473 bp, 46.12% G+C content), which consists of 18 scaffolds.

Gene prediction and annotation of the assembled genome were performed using the Rapid Annotations using Subsystems Technology (RAST) server (3) and the NCBI Prokaryotic Genome Annotation Pipeline (PGAP) (4). A total of 3,784 genes were predicted, including 3,390 coding sequences (CDS), 121 structural RNA encoding genes (102 tRNAs and 19 rRNAs), and 5 genes for noncoding RNA (ncRNA), and 268 genes were identified as pseudogenes. Various subsystems of essential functions, such as carbohydrate metabolism, amino acids and derivatives, protein metabolism, DNA and RNA metabolism, cell division and sporulation, etc., were mapped using RAST. Remarkably, the presence of several significant genes in this genome was observed, including the genes pertaining to acid bile resistance and adhesion to cells. This strain is also predicted to encode two putative bacteriocins and a siderophore, which might protect ZB29 and combat intestinal infections caused by drug-resistant pathogenic bacteria. The relevance of these genes in the draft genome validates the potential of ZB29 as a probiotic. In addition, this genome harbors 2 CRISPR arrays, which were further confirmed using CRISPRFinder (5).

However, exploring the genome of B. coagulans ZB29 to unveil the genetic determinants of safety and probiosis toward emerging challenges in commercialization still remains to be done.

### Data availability.

This whole-genome shotgun project has been deposited in DDBJ/EMBL/GenBank under the accession number QPFA00000000 (BioSample number SAMN09489659, BioProject number PRJNA475407, and SRA number SRR8204338). The version described in this paper is the first version (QPFA01000000).
